# Supplementary material for: A Multiplexed Urinary Biomarker Panel Has Potential for Alzheimer’s Disease Diagnosis Using Targeted Proteomics and Machine Learning
Source: Int J Mol Sci. 2023 Sep 6;24(18):13758. doi: 10.3390/ijms241813758 (PMC10531486; doi:10.3390/ijms241813758)
Supplement: Supplementary file 1 [file ijms-24-13758-s001.zip › ijms-2577840-supplementary.pdf]

Table S1. Significantly different proteins in the comparison of urine protein expression between AD and healthy controls. *The table shows the gene name, protein name, accession number and the p-value from Student's t-test between AD and controls.*

Gene	Protein name	Accession	p-value
ACTN4	Alpha-actinin-4	O43707	4.6E-02
ANXA3	Annexin A3	P12429	5.0E-02
APOA4	Apolipoprotein A-IV	P06727	1.3E-02
ATP6V1A	V-type proton ATPase catalytic subunit A	P38606	4.8E-02
B2M	Beta-2-microglobulin	P61769	2.5E-02
BCAS1	Breast carcinoma-amplified sequence 1	O75363	2.3E-02
BTD	Biotinidase	P43251	4.8E-02
BTN2A1	Butyrophilin subfamily 2 member A1	Q7KYR7	1.8E-02
CCDC126	Coiled-coil domain-containing protein 126	Q96EE4	8.8E-03
CD5L	CD5 antigen-like	O43866	4.5E-02
CEP162	Centrosomal protein of 162 kDa	Q5TB80	5.0E-02
CILP	Cartilage intermediate layer protein 1	O75339	1.2E-02
CP	Ceruloplasmin	P00450	2.8E-02
CYR61	CCN family member 1	O00622	4.1E-02
DSP	Desmoplakin	P15924	3.1E-02
ERVK-9	Endogenous retrovirus group K member 9 Rec protein	P63128	2.6E-02
FRMD4B	FERM domain-containing protein 4B	Q9Y2L6	1.5E-02
GPI	Glucose-6-phosphate isomerase	P06744	4.1E-02
HPX	Hemopexin	P02790	2.9E-02
KIF13B	Kinesin-like protein KIF13B	Q9NQT8	3.8E-02
LAIR1	Leukocyte-associated immunoglobulin-like receptor 1	Q6GTX8	5.0E-02
LASP1	LIM and SH3 domain protein 1	Q14847	3.4E-02
MAGIX	PDZ domain-containing protein MAGIX	Q9H6Y5	3.0E-02
METTL8	tRNA N	Q9H825	1.7E-02
MIEN1	Migration and invasion enhancer 1	Q9BRT3	4.2E-02
NAPSA	Napsin-A	O96009	2.2E-02
OLR1	Oxidized low-density lipoprotein receptor 1	P78380	2.8E-02
PHF6	PHD finger protein 6	Q8IWS0	4.5E-02
PLD3	5'-3' exonuclease PLD3	Q8IV08	1.5E-02
PLIN3	Perilipin-3	O60664	1.1E-02
PURG	Purine-rich element-binding protein gamma	Q9UJV8	4.0E-02
QSOX1	Sulfhydryl oxidase 1	O00391	4.6E-02
RAB1C	Ras-related protein Rab-35	Q92928	3.8E-02
REG1B	Lithostathine-1-beta	P48304	2.8E-02
RIOK3	Serine/threonine-protein kinase RIO3	O14730	4.5E-02
SERPINB4	Serpin B4	P48594	3.6E-02
SERPINC1	Antithrombin-III	P01008	3.0E-02
SLC2A5	Solute carrier family 2, facilitated glucose transporter member 5	P22732	3.6E-02
SSX7	Protein SSX7	Q7RTT5	3.7E-02
TSPAN14	Tetraspanin-14	Q8NG11	4.2E-02
TXNDC17	Thioredoxin domain-containing protein 17	Q9BRA2	9.1E-03
WTAP	Pre-mRNA-splicing regulator WTAP	Q15007	2.3E-03

Table S2. Analytical details of the peptides included in the targeted assay. *The precursor proteins of the peptides are represented as gene names. Amino acid sequences, the transitions of precursor to product ions and the cone and collision energies are presented.*

Gene	Amino acid sequence	Precursor [m/z]	Product #1/2 [m/z]	Collision voltage #1/2 [V]
A2M	AFQPFFVELTMPYSVIR	1023.0	1079.6/1208.6	37/37
ABCA7	ADTDMEGSVDTR	648.8	763.3/894.4	22/22
ABCA7	LLQDVGLVSK	536.3	717.4/845.5	17/17
ADIPOQ	IFYNQQNHYDGSTGK	591.3	666.3/1106.5	20/20
ANXA2	TNQELQEINR	622.8	659.3/772.4	22/22
ANXA2	TPAQYDASELK	611.8	825.4/1024.5	22/22
APOA4	LAPLAEDVR	492.3	589.3/799.4	17/17
APOA4	LLPHANEVSQK	412.6	456.7/505.3	14/14
APOE	LGPLVEQGR	484.8	588.3/701.4	17/17
APOE	AATVGSLAGQPLQER	749.8	642.4/827.6	18/26
APP	GLTTRPGSGLTNIK	472.3	572.3/622.9	16/16
B4GT1	YWLYYLHPVLQR	551.0	612.4/651.4	19/19
BCHE	YLTLNTESTR	599.3	707.3/921.5	21/21
BCHE	YGNPNETQNNSTSWPVFK	1042.0	490.3/874.9	37/37
BST1	SAAAATQR	388.2	475.3/617.3	13/13
BST1	GFFADYEIPNLQK	514.6	599.4/830.3	17/17
C3	TVMVNIENPEGIPVK	820.4	853.5/982.5	29/29
CAPN2	NFFLTNR	456.2	650.4/797.4	16/16
CCL4	NFVVDYYETSSLCSQPAVVFQTK	894.8	889.5/1104.6	27/27
CD5L	GQWGTVCDDGWDIK	818.9	1008.4/1107.5	29/29
CDKL5	FEILGVVGEGAYGVVLK	584.0	678.4/806.5	20/20
CDKL5	GTSMCPTLQVR	625.3	713.4/873.5	22/22
COL4A2	GLDGYQGPDGPR	616.3	541.3/726.4	22/22
COL4A2	SVSIGYLLVK	539.8	692.4/892.6	19/19
COL6A3	QLGTVQQVISER	679.4	859.5/1116.6	24/24
CP	EDEDFQESNK	620.8	752.4/867.4	22/22
CP	DNEDFQESNR	627.3	633.3/780.4	22/22
CRISPLD1	AAVHAGVVR	440.3	501.4/638.4	19/19
CRISPLD1	GNWWGHAPYK	405.9	407.3/478.3	18/18
CSF1R	VVEATAFGLGK	546.3	764.4/893.5	19/19
CST3	ALDFAVGEYNK	613.8	780.4/927.5	22/22
CYCS	TGPNLHGLFGR	390.2	505.8/534.3	13/13
DCXR	AVIQVSQIVAR	592.4	772.5/900.5	21/21
DKK3	SAVEEMEAEEAAAK	732.8	818.4/949.4	26/26
DKK3	EVPDEYEVGSFMEEVR	957.9	843.9/1053.5	34/34
DNAH1	LWYHENCR	393.2	449.2/578.3	18/18
DNAH1	ANLMAAYTGR	534.3	638.3/769.4	18/18
DNAH12	MHIFQPEISK	410.5	529.2/573.3	11/11
DNAH12	SFIAVPVTNR	552.3	586.3/756.4	14/14
EFNA5	VENSLEPADDTVHESAEPSR	728.0	755.8/820.4	25/25
EPO	TITADTFR	462.7	609.3/710.3	16/16

FABP5	TTQFSTLGEK	556.3	781.4/909.5	20/20
FABP5	ELGVGIALR	464.3	529.3/685.4	16/16
FGA	AQLVDMK	402.7	492.2/605.3	14/14
FGA	GLIDEVNQDFTNR	760.9	894.4/1237.5	27/27
GDA	YTFPAEHR	340.8	512.3/609.3	14/14
GDA	FQNIDFAEEVYTR	544.6	667.3/796.3	13/13
GOLM1	AVLVNNITTGER	643.9	790.4/904.4	23/23
GOLM1	DTINLLDQR	544.3	644.4/758.4	19/19
GRN	ENATTDLLTK	553.3	690.4/791.5	19/19
GRN	AVALSSSVMCPDAR	732.4	1022.4/1109.5	26/26
HPX	YYCFQGNQFLR	748.3	862.5/1009.5	27/27
HSCB	LAEAESEAAMK	575.3	836.4/965.4	20/20
HSCB	LVHPDFFSQR	415.9	537.3/709.4	14/14
HSP90AB1	SIYYITGESK	580.8	634.3/797.4	20/20
HSPA1L	VEIIANDQGNR	614.8	703.3/774.3	22/22
HSPA5	ITITNDQNR	537.8	747.3/860.4	19/19
HSPA8	DAGTIAGLNVLR	600.3	742.5/855.5	21/21
HSPD1	VTDALNATR	480.8	645.4/760.4	17/17
ICAM1	ASVSVTAEDEGTQR	725.3	834.4/1006.4	26/26
ICAM1	LLGIETPLPK	540.8	797.5/854.5	19/19
IGHA2	TPLTANITK	479.8	647.3/760.4	13/13
IGHA2	WLQGSQELPR	607.3	729.4/786.4	23/23
IL7	LNDLCFLK	511.8	680.4/795.4	18/18
ITIH2	VQFELHYQEVK	473.9	666.4/803.4	16/16
ITIH2	TEVNVLPGAK	514.3	698.4/797.5	18/18
MASP2	AGYVLHR	408.2	425.3/524.3	14/14
MASP2	WPEPVFGR	494.3	575.3/801.4	17/17
MIEN1	ASNGETLEK	474.7	676.4/790.4	16/16
MIEN1	EQYPGIEIESR	660.8	900.5/1063.5	23/23
MMP3	TYFFVEDK	524.8	637.3/784.4	18/18
MSN	EDAVLEYLK	540.3	552.3/665.4	19/19
MYO10	LYCFLDTDNVPK	742.9	788.4/901.5	26/26
MYO10	MPDQFDQAVVLNQLR	592.0	643.4/742.5	20/20
NCAM1	LEGQMGEDGNSIK	689.3	819.4/950.4	24/24
NCAM2	SFNATAER	448.2	476.2/661.3	15/15
NCAM2	EALNPETIEIK	628.8	829.4/943.5	20/20
NEFL	YEEEVLSR	512.8	603.3/732.4	18/18
NEFL	VLEAELLVLR	577.9	613.4/813.5	20/20
NEFM	FVEEIIEETK	618.8	732.4/990.5	22/22
NFKBIZ	ASGQAVDDFK	519.2	879.4/694.3	18/18
NLRP3	YLEDLEDVDLK	676.3	831.4/946.5	24/24
NPC2	LVVEWQLQDDK	686.9	932.4/1160.6	24/24
NRP1	ATHGGLGLR	441.3	515.4/572.4	19/19
NRP1	EWIQVDLGLLR	447.9	458.3/571.4	12/12
OLR1	QQAEEASQESENELK	573.9	719.4/848.4	19/19
PGAM1	AMEAVAAQGK	488.3	644.4/773.4	17/17

PGK1	VLPGVDALSNI	549.5	466.5/885.8	8/10
PKM	ITLDNAYMEK	599.3	755.3/791.4	21/21
PLAU	SDALQLGLGK	501.3	615.4/728.5	17/17
PLD3	LLISCWGHSEPSMR	558.3	667.3/723.8	19/19
PLD3	ALLNVVDNAR	542.8	574.3/787.4	19/19
PPP3CB	GLTPTGMLPSGVLAGGR	792.4	656.9/813.5	28/28
PTGDS	TMLLQPAGSLGSYSYR	872.4	989.5/1157.6	31/31
PTGDS	AQGFTEDTIVFLPQTDK	955.5	363.4/588.6	42/26
QSOX1	LEEIDGFFAR	598.8	712.3/954.5	21/21
QSOX1	LDVPVWDVEATLNFLK	930.0	766.4/935.5	33/33
REG1B	ISCPEGTNAYR	634.3	681.3/907.4	22/22
REG1B	WHWSSGSLVSYK	479.6	496.3/609.4	16/16
RSF1	EADGGGVGR	409.2	445.3/502.3	18/18
SCRG1	DVFFGPK	405.2	448.2/595.3	13/13
SERPINA1	LSSWVLLMK	538.8	603.4/876.5	19/19
SERPINA3	LYGSEAFATDFQDSAAAK	946.4	952.4/1053.5	34/34
SERPINF2	LGNQEPGGQTALK	656.8	674.4/771.4	23/23
SERPINF2	HQMDLVATLSQLGLQELFQAPDLR	908.7	500.1/1112.8	18/18
SERPING1	LVLLNAIYLSAK	659.4	765.5/992.6	23/23
SIRT2	YHLPYPEAIFEISYFK	673.0	786.4/933.4	18/18
SIRT2	VICLVGAGISTSAGIPDFR	645.0	534.2/704.3	17/17
SOD3	VTGVVLFR	445.8	534.3/690.4	15/15
SOD3	AGLAASLAGPHSIVGR	492.9	582.8/618.3	16/16
SPP2	DALSASVVK	445.3	503.3/590.4	15/15
SPP2	VNSQSLSPYLFR	705.9	695.4/782.4	25/25
TCHHL1	GEDVQLTEDQEQPAR	572.3	600.3/642.3	19/19
TCHHL1	EISQEGDEPAR	615.8	644.3/988.4	22/22
TFEB	IQELEMQAR	559.3	747.4/876.4	20/20
TFEB	EYLSETYGNK	602.3	798.4/911.4	20/20
TNFß	MHLAHSTLKPAAHLIGDPSK	531.8	331.2/619.4	18/18
TNNT3	DLMELQALIDSHFEAR	630.0	764.9/830.4	21/21
TOLLIP	LNITVVQAK	493.3	645.4/758.5	17/17
TOLLIP	GPVYIGELPQDFLR	802.4	775.4/1074.6	29/29
TUBA4A	AVFVDLEPTVIDEIR	858.5	942.5/1299.7	31/31
TXN	LEATINELV	501.2	474.1/528.2	9/9
UBC	TITLEVEPSDTIENVK	894.5	905.5/1002.5	32/32
UHRF1BP1L	DCNVIATK	460.7	531.4/645.4	16/16
VCAM1	NTVISVNPSTK	580.3	732.4/944.5	20/20
VCAM1	LHIDEMDSVPTVR	756.6	251.2/472.4	28/24
RSF1	DISTITGHR	333.8	470.3/583.4	14/14
Internal standards				
ALDOA	ALQASALK	404.8	496.3/624.4	14 / 14
RSU1	ALYLSDNDFEILPPDIGK	1014.3	633.4/746.4	36 / 36
C3	SSLSVPYVIVPLK	704.4	357.3/934.6	25 / 25
ENO1 (yeast)	GNPTVEVELTTEK	709.1	623.5/948.7	18 / 20
GSTO1	GSAPPGPVPEGSIR	664.4	556.8/1015.6	23 / 23

TSP1	TIVTTLQDSIR	627.4	940.5/1039.6	22 / 22
